# ARPC5 deficiency leads to severe early-onset systemic inflammation and mortality

**DOI:** 10.1242/dmm.050145

**Published:** 2023-07-21

**Authors:** Elena Sindram, Andrés Caballero-Oteyza, Naoko Kogata, Shaina Chor Mei Huang, Zahra Alizadeh, Laura Gámez-Díaz, Mohammad Reza Fazlollhi, Xiao Peng, Bodo Grimbacher, Michael Way, Michele Proietti

**Affiliations:** ^1^ Institute for Immunodeficiency, Center for Chronic Immunodeficiency, Medical Center, Faculty of Medicine, University of Freiburg, Breisacher Straße 115, 79106 Freiburg, Germany; ^2^ Spemann Graduate School of Biology and Medicine (SGBM), University of Freiburg, Albertstr. 19A, 79104 Freiburg, Germany; ^3^ Faculty of Biology, University of Freiburg, 79104 Freiburg, Germany; ^4^ Department of Rheumatology and Clinical Immunology, Hannover Medical School, Carl-Neuberg-Str. 1, 30625 Hannover, Germany; ^5^ RESIST – Cluster of Excellence 2155, Hannover Medical School, Carl-Neuberg-Str. 1, 30625 Hannover, Germany; ^6^ Cellular Signalling and Cytoskeletal Function Laboratory, The Francis Crick Institute, London NW1 1AT, UK; ^7^ Immunology, Asthma and Allergy Research Institute, Tehran University of Medical Sciences, Tehran 1419733154, Iran; ^8^ Paediatrics Center of Excellence, Children's Medical Center, Tehran University of Medical Sciences, Tehran 1419733151, Iran; ^9^ Department of Genetic Medicine, Johns Hopkins University School of Medicine, Baltimore, MD 21218, USA; ^10^ Clinic of Rheumatology and Clinical Immunology, Center for Chronic Immunodeficiency (CCI), Medical Center, Faculty of Medicine, University of Freiburg, Hugstetterstraße 55, 79106 Freiburg, Germany; ^11^ DZIF – German Center for Infection Research, Satellite Center Freiburg, 79104 Freiburg, Germany; ^12^ CIBSS – Centre for Integrative Biological Signalling Studies, University of Freiburg, Schänzlestr. 18, 79104 Freiburg, Germany; ^13^ Department of Infectious Disease, Imperial College, London W2 1PG, UK

**Keywords:** Actin, Actinopathy, Arp2/3, Immunodeficiency, CRISPR/Cas9

## Abstract

The Arp2/3 complex drives the formation of branched actin networks that are essential for many cellular processes. In humans, the ARPC5 subunit of the Arp2/3 complex is encoded by two paralogous genes (*ARPC5* and *ARPC5L*) with 67% identity. Through whole-exome sequencing, we identified a biallelic *ARPC5* frameshift variant in a female child who presented with recurrent infections, multiple congenital anomalies, diarrhea and thrombocytopenia, and suffered early demise from sepsis. Her consanguineous parents also had a previous child who died with similar clinical features. Using CRISPR/Cas9-mediated approaches, we demonstrate that loss of ARPC5 affects actin cytoskeleton organization and function *in vitro*. Homozygous *Arpc5*^−/−^ mice do not survive past embryonic day 9 owing to developmental defects, including loss of the second pharyngeal arch, which contributes to craniofacial and heart development. Our results indicate that ARPC5 is important for both prenatal development and postnatal immune signaling, in a non-redundant manner with ARPC5L. Moreover, our observations add *ARPC5* to the list of genes that should be considered when patients present with syndromic early-onset immunodeficiency, particularly if recessive inheritance is suspected.

## Teaser

**Summary:** ARPC5 deficiency leads to a severe autosomal recessive primary immunodeficiency, featuring immune disease, multiple congenital anomalies and early postnatal death.

## INTRODUCTION

Conserved from yeast to man, the Arp2/3 complex is essential for a wide variety of fundamental cellular processes through its ability to generate branched actin filament networks (Goley and Welch, 2006). Though comprised of two actin-related proteins (Arp2 and Arp3) and five additional subunits (ARPC1-5), mammalian Arp3, ARPC1 and ARPC5 are each encoded by pairs of genes (*ACTR3*/*ACTR3B*, *ARPC1A/ARPC1B* and *ARPC5/ARPC5L*) that give rise to proteins with 91, 67 and 67% sequence identity, respectively (Balasubramanian et al., 1996; Jay et al., 2000; Millard et al., 2003). Previous studies have shown that the ARPC1 and ARPC5 paralogous pairs of proteins confer different actin-nucleating efficiencies to the Arp2/3 complex (Abella et al., 2016). Arp3- and Arp3B-containing complexes also show divergent properties (Galloni et al., 2021). This suggests that the mammalian Arp2/3 complex consists of eight iso-complexes that have evolved to perform distinct cellular or physiological functions.

In addition to the eight different possible iso-complexes, the Arp2/3 complex itself is subject to many layers of regulation. Amongst the most important are the Wiskott-Aldrich Syndrome protein (WASp) family of Arp2/3 activators, including N-WASp, Wave1, Wave2 and Wash (Alekhina et al., 2017; Gautreau et al., 2022). Mutations in WASP are known to cause Wiskott-Aldrich Syndrome (WAS), a complex systemic disorder combining immunodeficiency, autoimmunity, autoinflammation, atopies and predisposition to malignancy (Albert et al., 2011; Candotti, 2018; Rivers and Thrasher, 2017). Since the discovery of this seminal genotype-phenotype relationship, mutations in other proteins with important roles in actin cytoskeletal regulation have been found that lead to similar human diseases; together, these are often known as ‘immuno-actinopathies’ (Papa et al., 2020; Tur-Gracia and Martinez-Quiles, 2021).

One of the most recently identified immuno-actinopathies is the autosomal recessive ARPC1B deficiency. Mutations in this subunit of the Arp2/3 complex result in severe inflammation and immunodeficiency, as well as impaired cytotoxic T lymphocyte maintenance and cytolytic activity (OMIM: 617718) (Brigida et al., 2018; Kahr et al., 2017; Kuijpers et al., 2017; Leung et al., 2021; Papadatou et al., 2021; Randzavola et al., 2019; Somech et al., 2017; Volpi et al., 2019). ARPC1A, which is normally expressed at low levels in hematopoietic lineages, is substantially upregulated in the absence of ARPC1B but is unable to compensate for its loss of function (LOF) (Kahr et al., 2017; Kuijpers et al., 2017; Randzavola et al., 2019).

An *in vitro* system that recapitulates neonatal skeletal muscle development has also elucidated unique roles for ARPC5 isoforms (Roman et al., 2017). In this system, ARPC5L- but not ARPC5-containing Arp2/3 complexes are required for the correct positioning of nuclei in the periphery of myofibers, whereas ARPC5 but not ARPC5L is involved in the organization of transverse triads (Roman et al., 2017). More recently, the loss of ARPC5 isoforms in B16 mouse melanoma cells reveals that the absence of ARPC5 but not ARPC5L leads to slower cell migration and reduced Mena/VASP recruitment to the protruding leading edge ARPC5 isoforms (Fassler et al., 2023). ARPC5 isoforms that, according to available datasets (https://pax-db.org, http://www.proteinatlas.org), are ubiquitously expressed and also drive distinct Arp2/3-dependent nuclear and cytoplasmic actin assembly during CD4 T-cell activation (Sadhu et al., 2023). Although it is now clear that ARPC5 isoforms have differential cellular functions, their *in vivo* physiological roles remain to be established.

We now report the consequences of ARPC5 LOF in humans and mice. We show that human ARPC5 deficiency cannot be compensated for by the presence of wild-type (WT) ARPC5L, and leads to a syndrome featuring immune disease, multiple congenital anomalies and early postnatal death. Moreover, unlike ARPC1B mice, which do not show impaired prenatal development or survival (Kuijpers et al., 2017), constitutive ARPC5 LOF results in embryonic lethality. Thus, this gene should be included in genetic testing for families with recurrent fetal and/or infantile deaths, particularly if consanguinity is present.

## RESULTS

### Clinical presentation

**Fig. 1.**
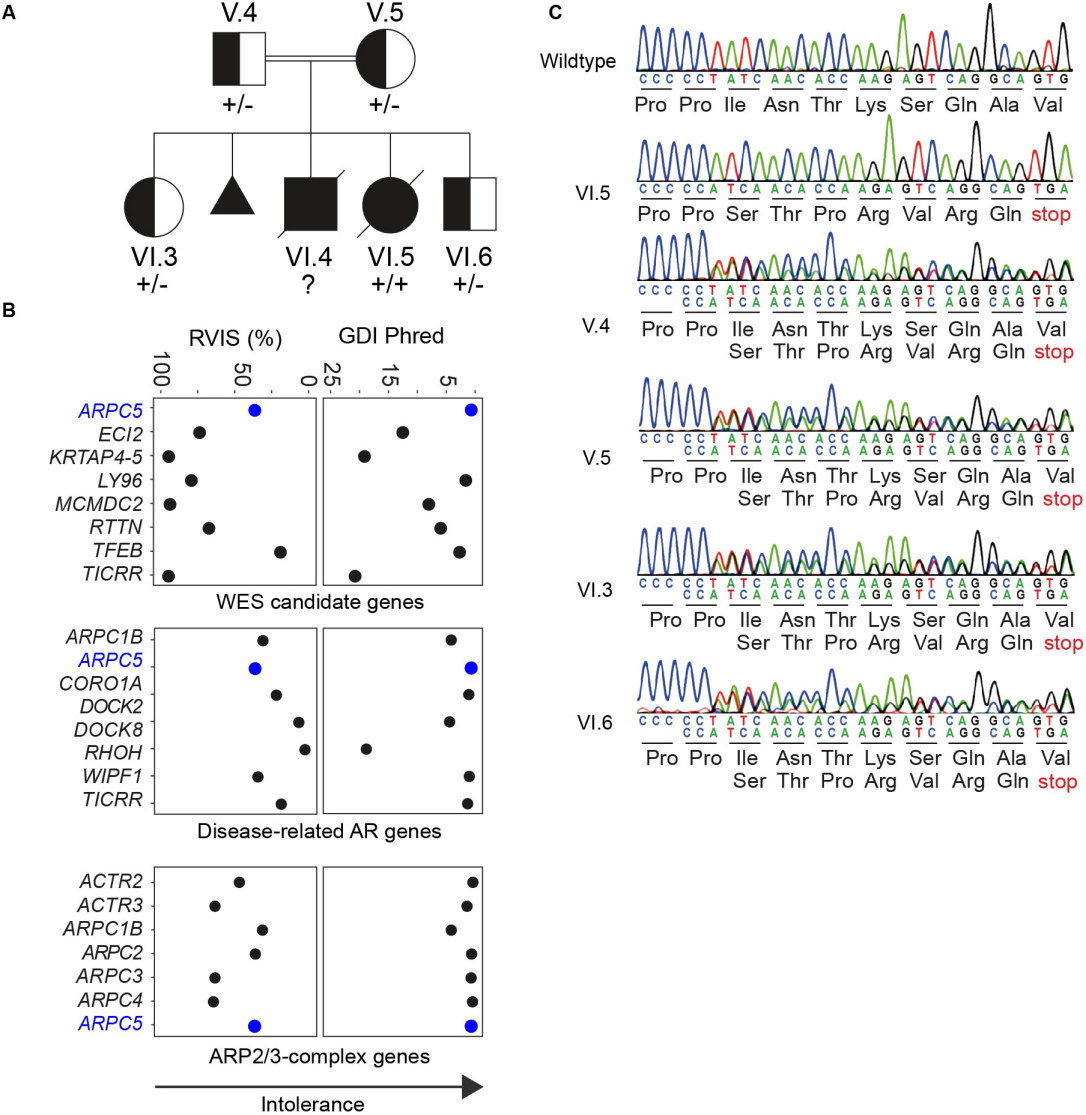
**ARPC5 deficiency manifests as a severe systemic autosomal recessive disorder.** (A) Index patients (VI.4 and VI.5) with parents and siblings. Complete family pedigree is available in Fig. S2. Half-filled symbols represent unaffected *ARPC5* heterozygous mutation carriers. Filled symbols represent affected homozygous (and presumed homozygous) carriers. (B) Intolerance scores (GDI, gene damage index; RVIS, residual variation intolerance score) for genes in which homozygous variants were identified during WES analysis of VI.5 (WES candidate genes) compared to the scores for genes that are already known to be associated with IEIs (Disease-related AR genes) and related to ARPC5, or encoding other subunits of the Arp2/3 complex (ARP2/3 complex genes). (C) Sanger sequencing electropherograms of the index family showing the single nucleotide deletion.

We studied the now-deceased son (VI.4) and daughter (VI.5) of consanguineous Iranian parents, who presented with multiple invasive infections and oral abscesses in early infancy (Fig. 1A; Tables 1 and 2). They also had two other healthy siblings – a 15-year-old sister (VI.3) and a 3-year-old brother (VI.6). Of note, the mother's second pregnancy resulted in a first trimester miscarriage (at 8 weeks); prenatal ultrasound could not identify heart formation in the fetus. VI.4 was found postnatally to have multiple congenital anomalies including a congenital heart defect (CHD) (patent foramen ovale), cleft palate and hypoplastic corpus callosum (Table 1). He subsequently developed hydrocephalus, severe pulmonary hypertension, oral ulcers and erosive gastritis. VI.5 was also born with CHD (moderate pulmonary stenosis and atrial septal defect) and developed oral ulcers as well as persistent diarrhea. Both patients also developed symptomatic thrombocytopenia at age 1 month (Table 2) and died of sepsis at age 3 months (Table 1). As far as we know, neither showed evidence of atopy or developed malignancy, but the increased risk for these potential complications may have been masked by their early deaths.

**Table 1.**
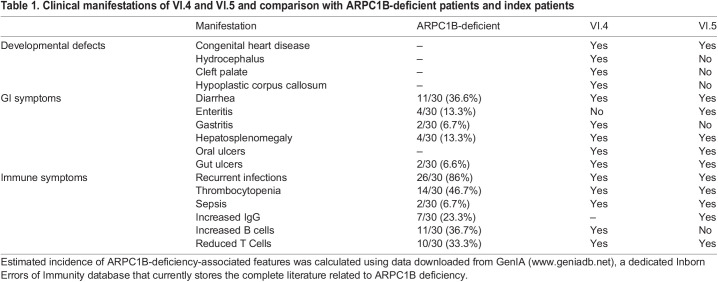
Clinical manifestations of VI.4 and VI.5 and comparison with ARPC1B-deficient patients and index patients

**Table 2.**
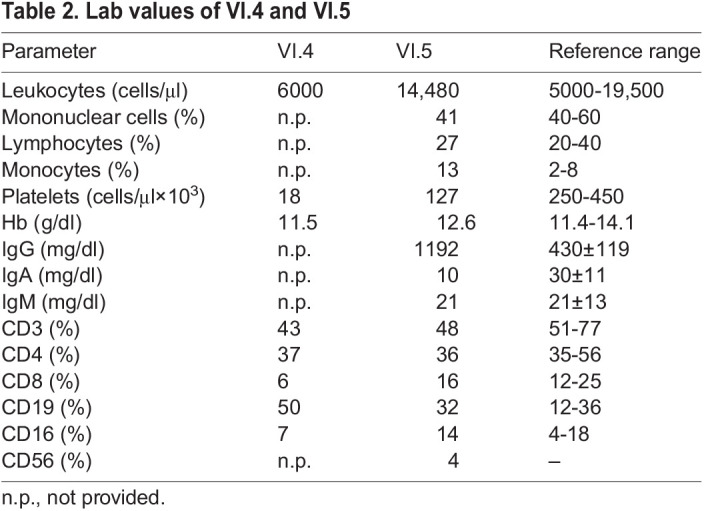
Lab values of VI.4 and VI.5

Laboratory studies for both affected siblings showed normal leukocyte counts, and reduced T-cell and platelet counts in comparison to age-matched controls (Table 2). VI.5 also had monocytosis, which may have been secondary to increased monocyte recruitment during infection; VI.4 showed increased B-cell numbers. The former also had elevated IgG levels, whereas IgA was slightly decreased compared to age-matched controls (Table 2). Unfortunately, more detailed additional immunophenotyping to investigate parameters such as lymphocyte subsets, functional responses to mitogens and antigens, serum cytokine levels, or markers of type I and II interferon and/or NF-κB activation was not possible due to the early deaths of both children.

### Identification of a homozygous truncating mutation in *ARPC5*

The known consanguinity of both unaffected parents was strongly suggestive of a recessively inherited defect. To confirm our hypothesis, we performed whole-exome sequencing (WES) using VI.5 as proband, because VI.4 had unfortunately already passed away with no stored DNA sample available. We first interrogated genes already known to be associated with inborn errors of blood and immunity for homozygous candidate variants but identified none of interest. Application of a stringent filtering strategy (Fig. S1A) identified eight rare homozygous candidate variants (Fig. S1B). We then further narrowed down this list via segregation testing on available family members, performing WES on the patient's parents, living siblings and 14 extended family members (Fig. S2). Trio analysis found that only four of the eight candidates were heterozygous in both parents and absent in homozygosity in unaffected individuals (Fig. S1B). Amongst these, we predicted that the identified LOF variant in *ARPC5* (ENST00000359856.11:c.189delT, p.Ile64Serfs*8) could lead to a severe clinical phenotype, via potentially damaging effects on ARPC5 protein, a subunit of the Arp2/3 complex. This was supported by the recent finding that LOF in ARPC1B, another member of the Arp2/3 complex (Kahr et al., 2017; Kuijpers et al., 2017; Volpi et al., 2019), led to some overlapping clinical features with our patients (Table 1). This variant was also notably absent from large population databases such as gnomAD exomes or genomes, TopMED/BRAVO, All of Us, Exome Sequencing Project or the 1000 Genomes Project, and large variant databases such as ClinVar or LOVD. Moreover, it was the only gene harboring a variant with predicted high impact on protein function (frameshift) (Fig. 1C). In particular, gene mutation intolerance scores based on different algorithms [gene damage index (GDI; Itan et al., 2015) and residual variation intolerance score (RVIS; Gussow et al., 2016)] both found *ARPC5* to be amongst the top one or two most intolerant to functional variation of the candidate genes (Fig. 1B). Importantly, unaffected family members, including the parents and unaffected elder sister (VI.3), were at most heterozygous carriers (Fig. 1C; Fig. S2), while four additional relatives were also unaffected. At the time of our analysis, the mother was again pregnant and prenatal screening was performed on the fetus (VI.6), who was found to be a heterozygous carrier and carried to term (Fig. 1C; Fig. S2). This son is now 3 years old and healthy, which further supports our hypothesis that biallelic *ARPC5* LOF led to the clinical problems experienced by the deceased siblings. The clinical phenotype presented some clinical overlap with DiGeorge syndrome (which, in the majority of the cases, is caused by heterozygous chromosome 22 deletion); however, coverage analysis did not show signs of possible chromosome 22 (22q11) deletion (Fig. S3).

### *ARPC5*: p.Ile64Thrfs*8 leads to loss of ARPC5 and upregulation of ARPC5L

**Fig. 2.**
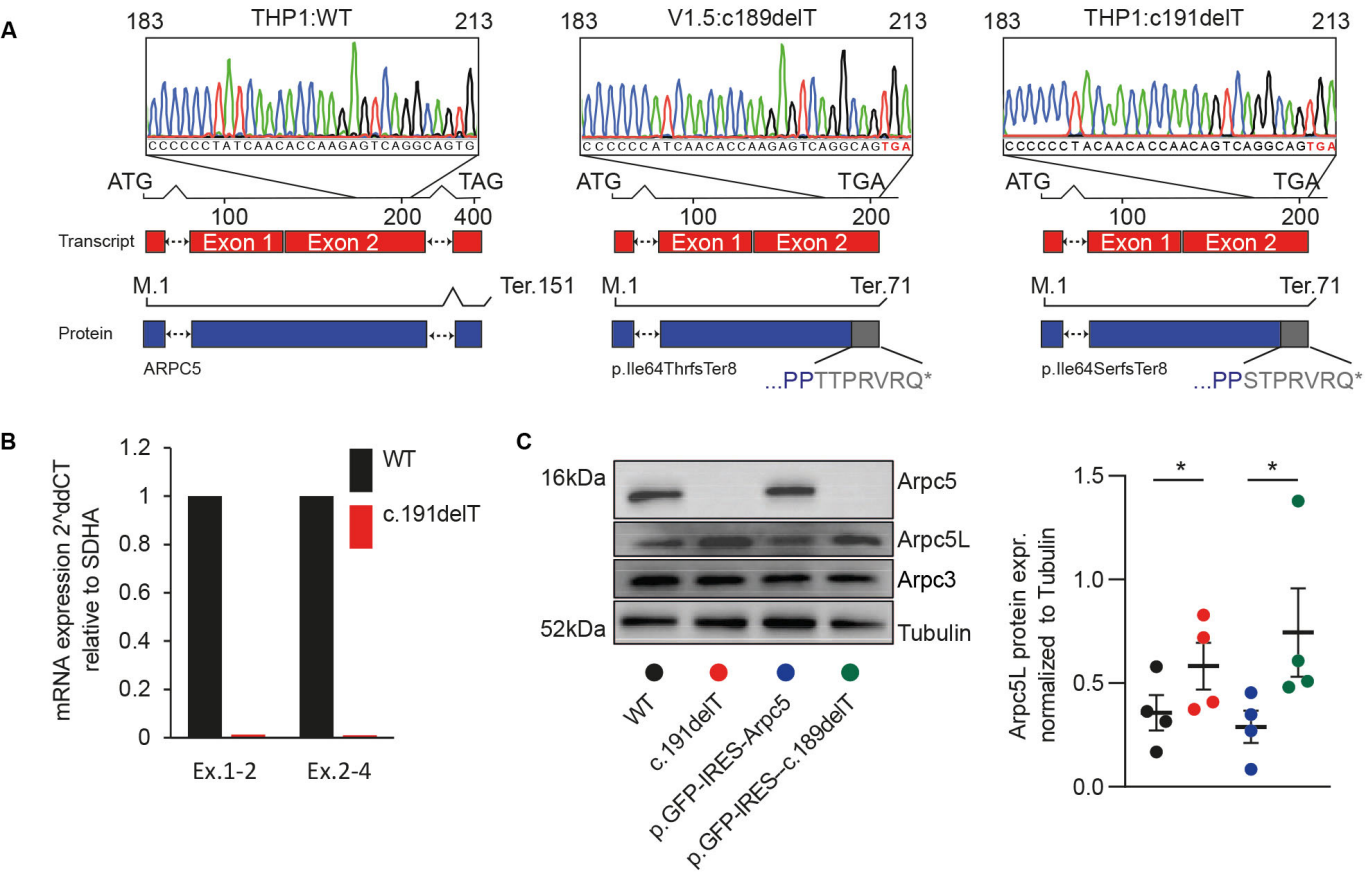
ARPC5:p.Ile64Thrfs*8 leads to loss of ARPC5 and upregulation of ARPC5L. (A) Schematic of mRNA and Sanger sequencing of guide DNA from WT THP1 cells, the index patient VI.5 and c.191delT-THP1cells. (B) mRNA quantification of ARPC5 exons 1-2 mRNA (Ex.1-2) and exons 2-4 (Ex.2-4) in WT and c.191delT-THP1 cells. (C) Immunoblot analysis of ARPC5 and ARPC5L in WT, c.191delT-THP1 and c.191delT-THP1 cells reconstituted with WT or c.189delT ARPC5 cDNA. The graph shows the relative quantification of four independent replicates. Data are mean±s.e.m. *P<0.05 (paired two-tailed *t*-test).

The homozygous variant in *ARPC5* is a single nucleotide deletion (ENST00000359856.11:c.189delT, p.Ile64Serfs*8) in exon 2 predicted to result in the formation of a premature stop codon (Fig. 2A). The resulting RNA transcript would be expected to undergo nonsense-mediated decay, or potentially result in expression of a truncated protein containing only the first 66 amino acids of the native 151 residue protein (ENSP00000352918.6) as well as seven additional residues (TTPRVRQ) before the stop codon (Fig. 2A). Such a truncated protein would not assemble into the Arp2/3 complex, as the C-terminal half of ARPC5 is required to bind the rest of the complex (von Loeffelholz et al., 2020). To examine the functional consequences of the identified variant, we targeted the same exon of the *ARPC5* gene affected by the identified c.189delT mutation via CRISPR/Cas9 in THP1 cells. We generated a c.191delT variant which also leads to a frameshift variant (p.Ile64Serfs*8) as observed in the patient with the same additional residues, with the exception that the first threonine residue is now a serine (STPRVRQ) (Fig. 2A). This variant resulted in the loss of ARPC5 expression both at mRNA and protein levels (Fig. 2B,C). In contrast to the WT allele, transduction with lentiviral vectors expressing the ARPC5-c.189delT allele failed to rescue ARPC5 expression in ARPC5-CRISPR/Cas9-depleted (c.191delT) THP1 cells. Interestingly, upregulation of the ARPC5L isoform was seen in the absence of ARPC5 (Fig. 2C). Our observations confirm that the c.189delT mutation abrogates ARPC5 mRNA and protein expression and that loss of ARPC5 leads to ARPC5L upregulation.

### ARPC5 deficiency affects actin cytoskeleton organization and function

**Fig. 3.**
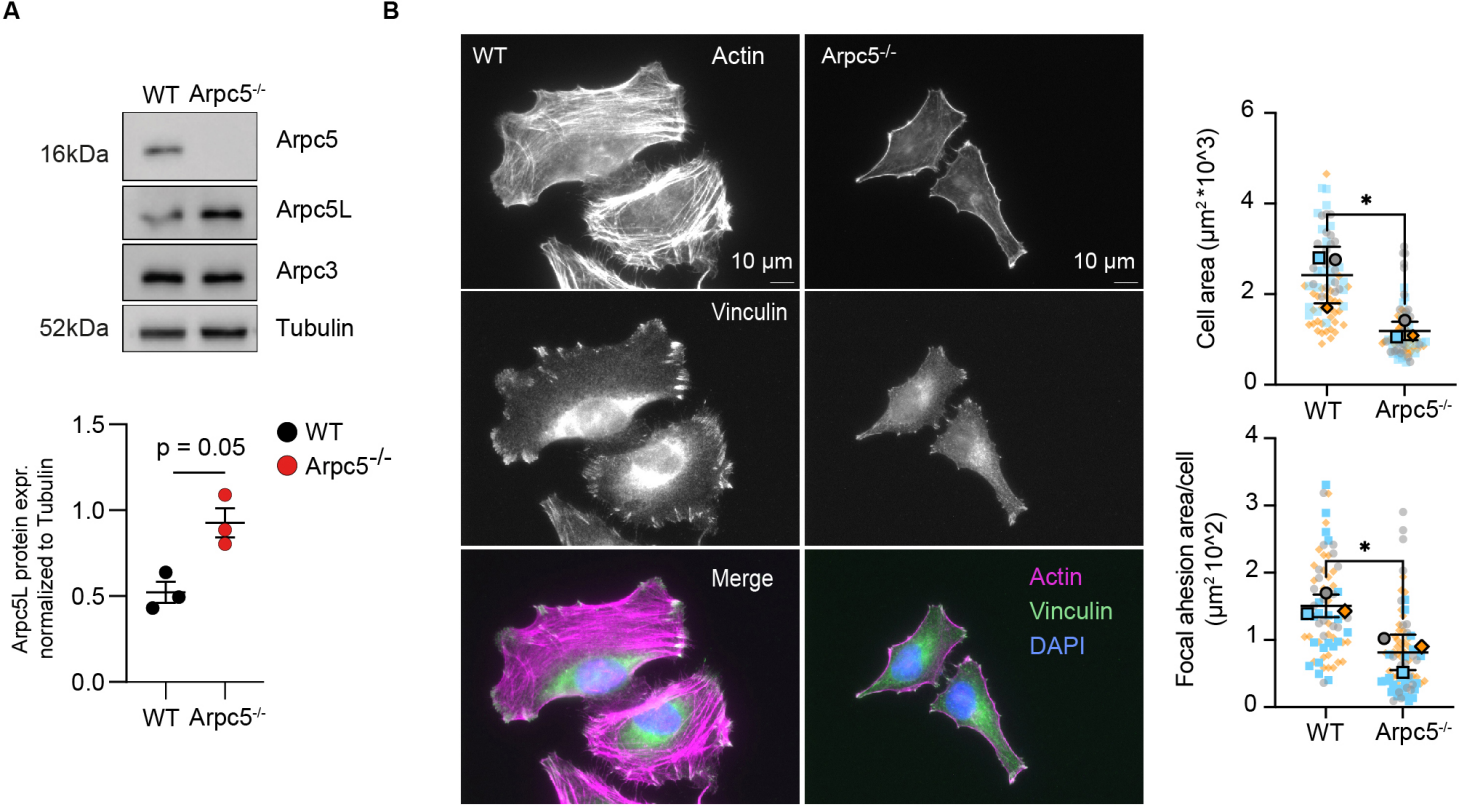
**Arpc5 deficiency affects cell spreading, actin stress fiber morphology and focal adhesions.** (A) Immunoblot analysis of ARPC5L in WT and ARPC5^−/−^ HeLa cells (top). Quantification and statistical analysis of independent triplicates (bottom). Data are mean±s.e.m. *P<0.05 (paired two-tailed *t*-test). (B) Immunofluorescence images of HeLa WT and ARPC5^−/−^ cells labeled with Alexa Fluor 488 Phalloidin (actin, magenta), Vinculin (focal adhesions, green) and DAPI (DNA, blue) (left). Quantification of cell and focal adhesion area of WT and ARPC5 KO HeLa cells (right). Three independent experiments were analyzed. *n*=20-30 cells per experiment. Data are mean±s.d. *P<0.05 (unpaired two-tailed *t*-test).

**Fig. 4.**
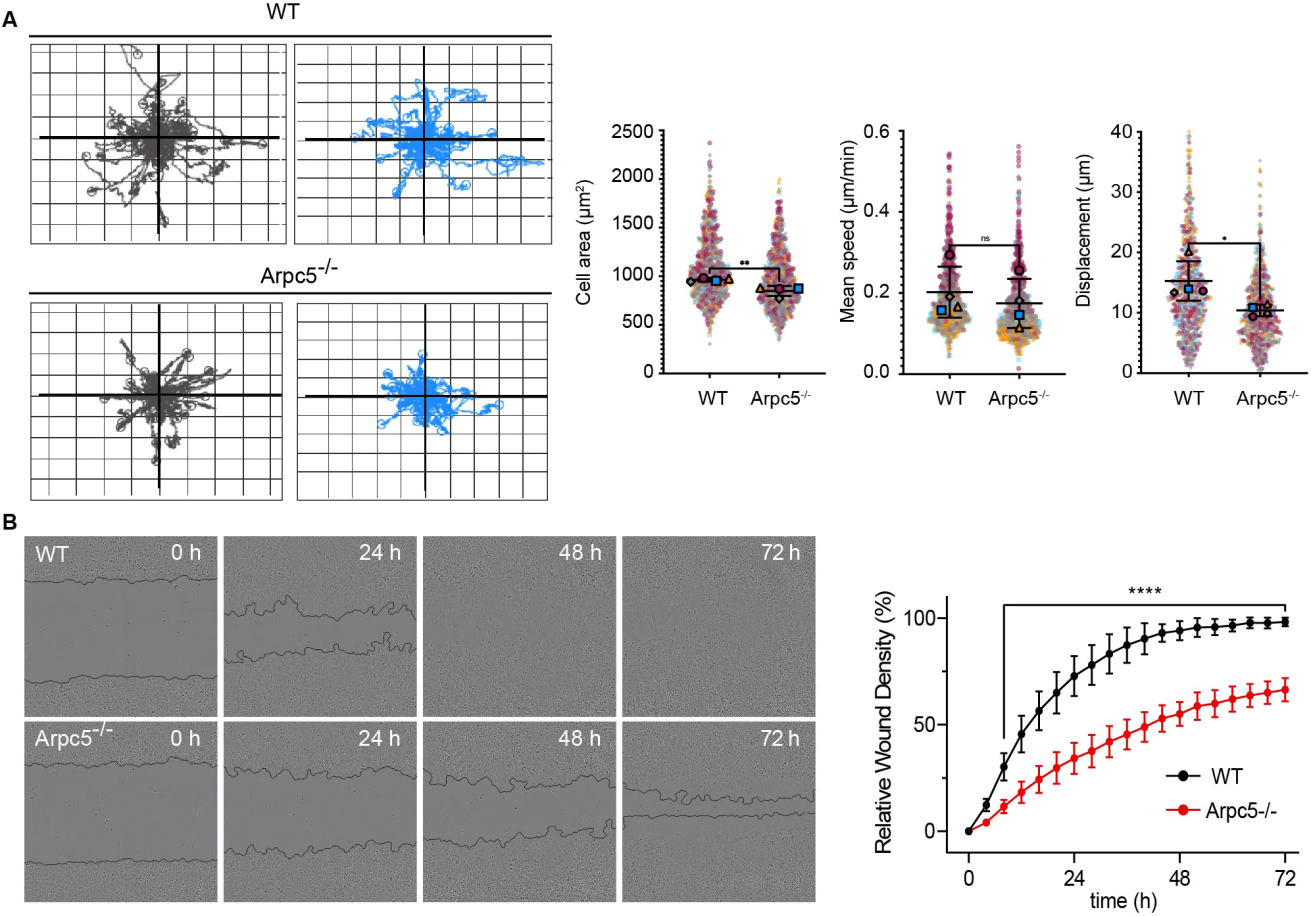
**Arpc5 deficiency affects cells migration.** (A) Representative spider graphs illustrating the displacement of WT or Arpc5^−/−^ Hela cells during 24-h random cell migration. Fifty cell tracks are shown in each graph (left). Gray and blue graphs show representative displacement tracks from two independent experiments. Quantification of cell area, speed and displacement of WT and Arpc5^−/−^ Hela cells during random migration (right). Four independent experiments were analyzed. *n*>200 cells per experiment. Data are mean±s.d. *P<0.05, **P<0.01 (unpaired two-tailed *t*-test). ns, not significant. (B) Representative phase images of HeLa WT and ARPC5^−/−^ cells are shown for the indicated times in hours after the scratch (left). Relative wound density of four technical replicates for each time point is shown (right). Data are mean±s.d. ****P<0.0001 (two-way ANOVA).

To investigate whether ARPC5L compensates for the lack of ARPC5, we examined the impact of ARPC5 deficiency on the organization and function of the actin cytoskeleton in HeLa cells. To do this, we generated HeLa *ARPC5^−/−^* cells using CRISPR/Cas9. As observed in THP1 cells (Fig. 2C), loss of ARPC5 in HeLa cells resulted in increased ARPC5L expression (Fig. 3A). Nevertheless, there was a dramatic reduction in cell spreading together with a loss of actin stress fibers and focal adhesions (Fig. 3B). Moreover, the same *ARPC5*^−/−^ HeLa cell clone had reduced cell migration compared to WT controls, with mean±s.d. velocities and displacements of 0.17±0.06 μm/min and 10.39±0.94 μm versus 0.20±0.06 μm/min and 15.27±3.26 μm, respectively (Fig. 4A). Consistent with this, *ARPC5*^−/−^ cells were also significantly impaired in their ability to migrate into mechanically generated scratches relative to WT cells after 8 h. By 72 h, the WT cells had achieved 100% gap closure, whereas the *ARPC5*^−/−^ cells failed to attain even 75% (Fig. 4B). Loss of ARPC5 also delayed migration of adherent MDA-MB-231cells (a triple-negative breast cancer line) (Fig. S4A,B).

**Fig. 5.**
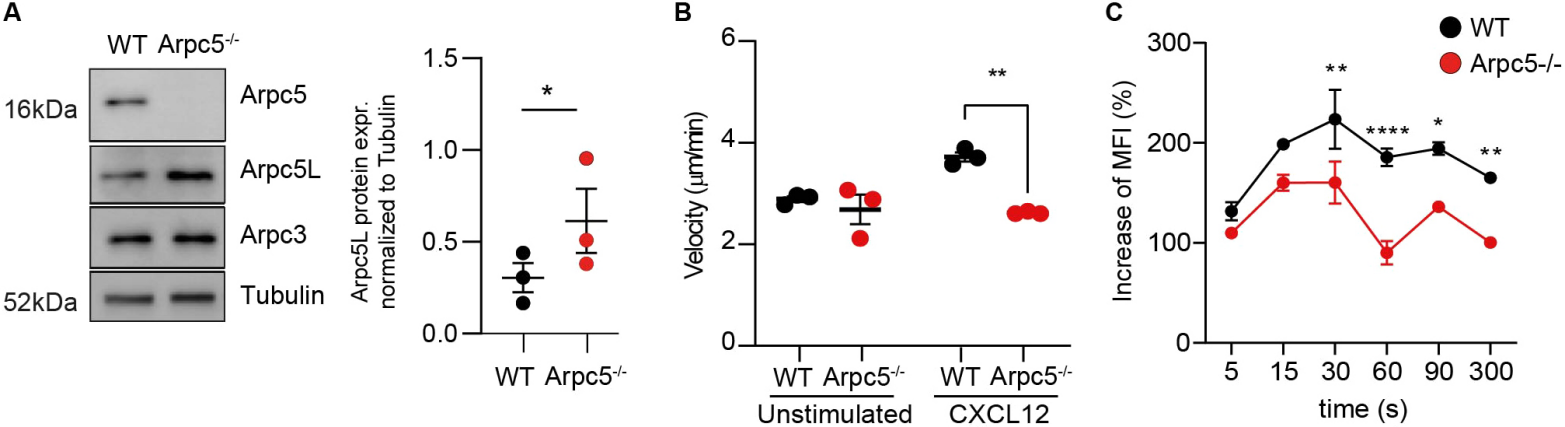
**Arpc5 deficiency impairs cell migration and actin polymerization of Jurkat cells.** (A) Immunoblot analysis of ARPC5L in WT and ARPC5^−/−^ Jurkat cells (left). Quantification and statistical analysis of independent triplicates (right). Data are mean±s.e.m. *P<0.05 (paired two-tailed *t*-test). (B) Mean velocity of WT and ARPC5^−/−^ Jurkat cells with and without CXCL12 treatment. Three independent experiments, >160 cells per experiment were analyzed. Data are mean±s.e.m. **P<0.01 (one-way ANOVA with Tukey's multiple comparison test). (C) F-actin polymerization in Jurkat cells with or without ARPC5 at the indicated time point after stimulation with CXCL12. MFI, mean fluorescence intensity. Three independent experiments were analyzed. Data are mean±s.d. *P<0.05, **P<0.01, ****P<0.0001 (two-way ANOVA).

Having observed defective cell migration of adherent HeLa *ARPC5*^−/−^ cells, we also decided to examine the impact of ARPC5 deficiency on non-adherent Jurkat cell migration under agarose. ARPC5 deficiency was again associated with increased ARPC5L protein levels (Fig. 5A). Unlike WT cells, the migration velocity of *ARPC5*^−/−^ cells did not increase after stimulation with CXCL12 (Fig. 5B). Interestingly there was a significant impairment in the extent of actin assembly after CXCL12 stimulation in *ARPC5*^−/−^ cells relative to WT controls, though the temporal kinetics appeared to be unaffected (still peaked at 30 s) (Fig. 5C). The same Jurkat *ARPC5*^−^/− cell clone was used in all the experiments shown in Fig. 5. These findings were also seen for the c.191delT-THP1cells (Fig. S4C). Taken together, we found that ARPC5 deficiency leads to reduced cell migration due to defects in actin organization and assembly that are not compensated for by an increase in ARPC5L expression.

### Defective organogenesis and embryonic lethality in *Arpc5* KO mice

**Fig. 6.**
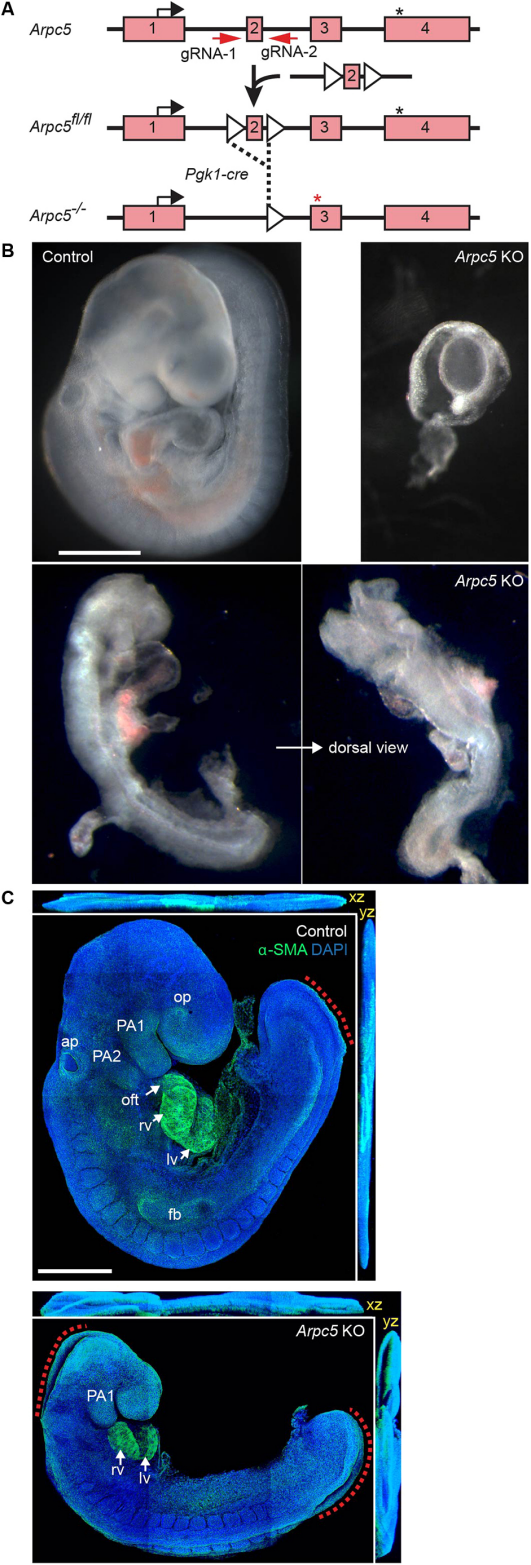
**Arpc5 deficiency in mice results in defective organogenesis and embryonic lethality.** (A) Schematic of the genomic organization of the Arpc5 locus highlighting the four exons (pink rectangles) as well the position of the start (black arrow) and stop (black asterisk) codons. The strategy used to generate the conditional KO mice and the position of two loxP sites (white triangles) flanking exon 2 are indicated. Cre-mediated removal of exon 2 results in a frameshift within exon 3 and a new stop codon (red asterisk). (B) Representative images of control and *Arpc5* KO embryos. Most *Arpc5* KO embryos are resorbed earlier (right) but a few show growth arrest and die by 9.5 dpf (bottom shows side and dorsal views). Scale bar: 1 mm. (C) Maximum intensity projection images and orthoview (*xz*, *yz*) of control and *Arpc5* KO embryos stained with α-smooth muscle actin (α-SMA, green) and DAPI (blue) at 9.5 dpf. Red dotted lines indicate partial closure of spinal neuropore (control and *Arpc5* KO) and cranial neuropore (*Arpc5* KO only). The second pharyngeal arch (PA2) and the cardiac outflow tract (oft) are absent from *Arpc5* KO embryos. The first pharyngeal arch (PA1), right (rv) and left (lv) ventricular myocardium, auditory pit (ap), optic pit (op) and forelimb bud (fb) are indicated where present. Scale bar: 0.5 mm.

To further investigate and characterize the effects of ARPC5 deficiency during mammalian development, we generated *Arpc5* knockout (KO) mice by targeting endogenous exon 2 with a floxed allele to inactivate the *Arpc5* gene using Cre recombinase expression (Fig. 6A; Fig. S5). The recombination was mediated by Pgk1-cre mice from as early as the one-cell zygote stage (Lallemand et al., 1998). Based on our observations in cells, deletion of exon 2 is expected to result in loss of *Arpc5* rather than expression of a truncated protein. The genomic deletion was verified by PCR (Fig. S5). Both *Arpc5*^+/+^ and *Arpc5*^+/−^ mice developed normally through all stages until adulthood, with the correct expected frequencies (Table 3). However, *Arpc5* homozygous LOF resulted in embryonic lethality, as no *Arpc5^−/−^* mice were identified from mating heterozygous *Arpc5*^+/−^ parents (Table 3). Microdissection followed by genotyping indicated that *Arpc5^−/−^* embryos only become distinct from controls at 8.5-9.5 days post-fertilization (dpf), and not earlier, and remain viable up to 9.5 dpf (Table 3) (Theiler, 1989). The majority of *Arpc5* KO embryos at 8.5-9.5 dpf are seen as tissue debris, or occasionally with abnormal morphology (4 out of 26 littermates) suggesting failure of post-implantation embryonic development in the absence of *Arpc5* (Fig. 6B). Immunofluorescence analysis revealed that control embryos at 9.5 dpf are fully turned and have developed 19-20 somite pairs, a circulatory system including a primitive heart and the presence of blood cells, the first and second pharyngeal arches (PA1 and PA2), and the closed cranial (anterior) neuropore, which are all hallmarks of Theiler stage 14 (Fig. 6C). *Arpc5* KO embryos show only 14-15 somite pairs, incomplete cranial neuropore closure, heart and PA2 formation, and have not yet completed embryonic turning. Thus, while axial patterning appears to be grossly intact, early global *Arpc5* LOF significantly disrupts tissue morphogenesis in mouse embryos, leading to post-implantation lethality.

**Table 3.**
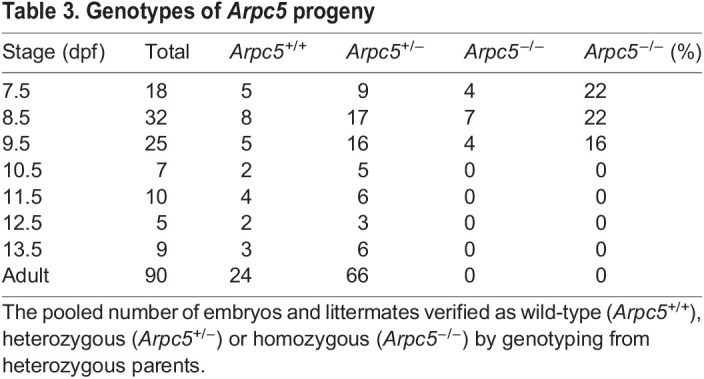
Genotypes of *Arpc5* progeny

## DISCUSSION

Herein, we describe human ARPC5 deficiency as a novel syndromic disorder characterized by severe, early-onset infections indicative of an inborn error of immunity (IEI), along with multiple congenital anomalies involving neurological, cardiovascular, craniofacial and hematopoietic development. Although we could only perform genetic analysis on one of the two affected patients (VI.5), multiple lines of evidence support the homozygous *ARPC5* c.189delT, p.(Ile67Serfs*8) variant we identified as being causative of the severe clinical phenotypes of our index patient and likely also her brother (VI.4) (Casanova et al., 2014): (1) the variant is sufficiently rare in control individuals, being absent from databases of either known variants or putatively health individuals; (2) it was not only found in homozygosity in a child of consanguineous parents, but also segregated appropriately with clinical presentation within a large family pedigree; (3) multiple lines of computational evidence support its exerting a deleterious effect on the gene or gene product; (4) the variant is a predicted nullomorphic allele in a gene encoding one subunit of an obligate complex where LOF of another human subunit (ARPC1B) has been previously shown to lead to similar disease phenotypes and mechanisms; (5) ARPC5 deficiency affects actin cytoskeleton organization and cell migration *in vitro*; (6) ARPC5 deficiency in a mouse model is associated with developmental phenotypes involving some of the same tissues/lineages as the human disease (Casanova et al., 2014). Because of the largely overlapping clinical phenotype, we believe that VI.4 also died because of an ARPC5 deficiency. However, given the lack of available material, we cannot exclude that the patient was heterozygous for the mutation and died from an additional autosomal recessive genetic defect.

Both genotype- and phenotype-driven inquiries identified *ARPC5* as our top candidate, but we did also consider candidate homozygous variants in seven other genes (Fig. S1B). Although these were potentially consistent with our hypothetical inheritance pattern, our index patient's presentation was less consistent with the expression and function of six of these genes than with that of *ARPC5*. Although human ARPC5 deficiency has never been previously reported in the literature, deficiency of ARPC1B, another subunit of the Arp2/3 complex, has been previously described – these patients show milder but similar clinical presentations as the patient described in this study (Kahr et al., 2017; Kuijpers et al., 2017; Volpi et al., 2019). In particular, both ARPC5- and ARPC1B-deficient patients share hallmarks of immunodeficiency and immune dysregulation as reflected in their histories of repeated respiratory and gastrointestinal infections, cytopenias, hypergammaglobulinemia, hepatosplenomegaly and enteropathy.

We did not have access to patient-derived cells, but were able to use CRISPR/Cas9-based methods to model the familial variant in THP1 cells, showing that it resulted in loss of both ARPC5 mRNA and protein expression. Of note, the absence of ARPC5 impacted the organization and function of the actin cytoskeleton despite the associated upregulation of its paralog ARPC5L. Similar results have also recently been observed in B16 mouse melanoma and Rat2 cells (Fassler et al., 2023). ARPC5L shares 67% homology with ARPC5 (Millard et al., 2003). However, the N-terminal half of ARPC5L is partially disordered compared to ARPC5 (von Loeffelholz et al., 2020) and this structural distinction may be an important contributor to ultimate differences in Arp2/3 complex activity when assembled with one or the other of the paralogs. This paradigm has been similarly illustrated for the mammalian ARPC1 paralogs (Abella et al., 2016). Similar to what we observed for ARPC5/ARPC5L, ARPC1A becomes substantially upregulated in the absence of ARPC1B (Brigida et al., 2018; Kahr et al., 2017; Kuijpers et al., 2017; Volpi et al., 2019); however, it is clearly insufficient to compensate for loss of the biological functions of ARPC1B in human cells. All of these data and our current results provide support for distinct biological roles of these paralogs, even within the context of the same complex, confirming that the Arp2/3 complex in higher eukaryotes is actually a family of eight iso-complexes with different properties.

The defective embryonic turning, incomplete cranial neuropore closure, and incomplete heart and PA1-2 formation seen in *Arpc5^−/−^* mice, as well as the developmental abnormalities observed in our patient, suggest a role for ARPC5 in mesoderm and neuroectoderm-derived organogenesis in higher eukaryotes. Pharyngeal arch mesoderm cells contribute substantial parts of the pharyngeal muscles and the primitive heart (Tzahor and Evans, 2011). At 8.0 dpf the PA1 mesoderm progressively migrates into the heart tube (i.e. future ventricular myocardium), while PA2 mesoderm gives rise to the cardiac outflow tract and primitive atria during heart-looping at 8.5-9.0 dpf (Fig. 6C). The migration of PA2 mesoderm towards the outflow tract is also influenced by co-migrating neural crest cells from the cranial neuropore, the signaling of which may be compromised by the lack of ARPC5. Consequently, the heart and the outflow tract development in KO embryos is limited. Collectively, Arpc5 mutation in mice abrogates tissue morphogenesis such as neural tube and the heart, followed by post-implantation lethality. The observed severe embryonic phenotype seen in our mouse model differs from the embryonic outcomes reported for mouse models of other core or unique subunits of the Arp2/3 complex. For example: (1) Arp3 is required for early preimplantation embryonic development (Vauti et al., 2007); (2) *Arpc3* LOF results in defective trophoblast outgrowth (Yae et al., 2006), whereas no embryonic lethality results from *Arpc1b* deficiency (Kuijpers et al., 2017). The lack of paralogs for some members of the complex and distinct developmental expression patterns for different isoforms may both contribute to these dissimilar phenotypes, but this hypothesis awaits further experimental validation.

Moreover, there is an expanding and already significant body of evidence implicating immune dysfunction in human prenatal complications and losses. However, few definitive links have been established thus far between prenatal anomalies or susceptibility to recurrent pregnancy loss and monogenic etiologies of postnatal IEIs (Carneiro-Sampaio et al., 2022). It is not surprising that ARPC5 deficiency joins other syndromic etiologies with known multi-lineage developmental impact. However, it is interesting to note that innate or adaptive immune dysregulation resulting in autoinflammation or autoimmunity are significant and shared features across almost all the other IEIs known to be associated with significant fetal or perinatal consequences (Carneiro-Sampaio et al., 2022). Moreover, many of these other IEIs also share abnormalities of hematopoiesis with ARPC5 deficiency – most notably, gain of function (GOF) in SAMD9L (Tesi et al., 2017), which itself has a close paralog in SAMD9. SAMD9 and SAMD9L are cytoplasmic proteins that are involved in regulating cell growth, proliferation and differentiation, particularly of hematopoietic cells in the bone marrow (Narumi, 2022). Though human disorders involving SAMD9 and SAMD9L mutations show overlapping clinical features, they also suggest non-redundant functions for these two paralogs. Similarly, mutations in the evolutionarily and functionally related proteasomal subunits also lead to syndromic conditions featuring predominantly hematologic, inflammatory and/or neurologic phenotypes (Kury et al., 2017; Torrelo, 2017; Ansar et al., 2020; Kroll-Hermi et al., 2020).

The observations we have presented in this study highlight some of the most exciting emerging topics linking basic science and clinical care today. Our findings further strengthen the role of actin cytoskeletal regulation in regulation of immune responses. We further add to the emerging repertoire of monogenic causes associated with both increased risk for fetal loss and significant postnatal hematopoietic and immune problems. From a basic science perspective, our observations further our paradigmatic understanding of how paralogous human genes evolve into overlapping but distinct essential roles, via structural divergence and spatio-temporal partitioning, enabling endless functional diversity to be achieved whilst preserving an elegant molecular parsimony.

## MATERIALS AND METHODS

### DNA isolation and sequencing

DNA extraction from peripheral blood samples was performed as previously described (Tesi et al., 2017). WES libraries were prepared using the SureSelect Human All Exon V6 kit from Agilent and sequenced in a NovaSeq 6000 system from Illumina. Bioinformatic analysis was performed as previously described (Rojas-Restrepo et al., 2021). The initial strategy for variant filtering and prioritization of WES data was carried out as previously described (Rojas-Restrepo et al., 2021), limiting the analysis to IEI-associated genes. A second (more stringent) filtering strategy was performed as shown in Fig. S2A. Segregation analysis was performed using polymerase chain reaction (PCR) after confirming the WES result. Direct sequencing was performed on an ABI 3730XL genetic analyzer (Applied Biosystems).

### Cell culture

THP1 cells (ATCC, #TIB-202™) and Jurkat cells (Clone E6-1, ATCC, #TIB-152™) were grown in RPMI 1640 10% fetal bovine serum (FBS), 2 mM L-glutamine, 1% penicillin-streptomycin (P/S). HeLa cells (ATCC, #CRM-CCL-2™) and MDA-MB-231 cells (ATCC, #HTB-26™) were cultured in Dulbecco's Modified Eagle Medium (DMEM), 10% FBS, 2 mM L-glutamine, 1% P/S. The cells were maintained at a density of 0.5×10^6^ cells/ml, changing the medium every 2-3 days.

### CRISPR-Cas9 knockout of ARPC5

CRISPR-Cas9 technology was used to generate ARPC5 KO Jurkat, THP1, HeLa and MDA-MB-231 cells. As the identified mutation is predicted to be LOF, we decided for a genetic strategy aimed at generating insertion and/or deletion in exon 2 via CRISPR/Cas9. The GAAAGGACGAAACACCGCTGACTCTTGGTGTTGATAGGGTTTTAGAGCTAGAAATAGCA guide targeting exon 2 of ARPC5 was designed using the online tool CHOPCHOP (https://chopchop.cbu.uib.no/) to target c.191T. We chose this guide RNA as it had a KO efficiency of ∼50% and a GC content of 45%. We predicted that DNA double-strand break repairing by non-homologous end joining and the consequent deletion of one base pair at the expected cut site of 191 could result in the same frameshift as the identified c.189delT mutation. The guide was cloned into the plasmid pMAX-Crispr with Cas9-2A-eGFP (provided by Dr Eva Bartok, Universitätsklinikum Bonn, Bonn, Germany) using Gibson assembly. Briefly, the vector was linearized using SwaI, the guide RNA (gRNA) was annealed at an equimolar ratio with the universal antisense oligo (GCCTTATTTTAACTTGCTATTTCTAGCTCTAAAAC) and the annealed oligos were filled in with Klenow fragment. Finally, the digested vector and the oligo were assembled using Gibson Assembly Master Mix (New England Biolabs, #E2611S). After transformation into Stbl3 chemically competent *Escherichia coli*, single colonies were grown in LB medium and plasmid DNA was extracted. Sanger sequencing was performed for confirmation. Cells of interest were electroporated using the Neon™ Transfection System: Jurkat (2×10^6^ cells, 10 µg, 1350 V, 10 ms, three pulses), THP1 (1.25×10^6^ cells, 20 µg, 1250 V, 50 ms, one pulse), HeLa (1.25×10^6^ cells, 10 µg, 1005 V, 35 ms, two pulses), MDA-MB-231 (0.95×10^6^ cells, 20 µg, 1400 V, 10 ms, four pulses). The following day, GFP cells were fluorescence-activated cell sorted at a density of 1 cell/well in a 96-well plate in RPMI/DMEM, 10% FBS, 2 mM L-glutamine. The growing clones were screened for KO by western blot. Clones with absent ARPC5 protein expression were confirmed by Sanger sequencing after PCR amplification of the target exon. Screening of ∼300 potential KO clones in several cell lines allowed us to identify a mutation that recapitulates the same stop codon as observed in the patient.

### Lentivirus transduction of ARPC5-KO cells

The ARPC5 WT complementary DNA (cDNA) (uniprot: O1551) and the ARPC5 cDNA containing the c.189del, p.Ile64Serfs*8 mutation (MUT) (provided by Dr Antonio Carusillo, Universitätsklinikum Freiburg, Freiburg, Germany), were cloned into pLenti-IRES-GFP-Puro plasmid. HEK 293T cells were transfected with pLenti-ARPC5 WT/MUT-IRES-GFP-Puro, psPAX2, and pMD2G to produce lentiviral particles. After 48 h, the supernatant was transferred to ARPC5 KO Jurkat, THP1, HeLa and MDA-MB-231 cells. Cells were incubated for 48 h and sorted for GFP expression.

### SDS-PAGE and western blotting

Cells were collected and lysed in 2× RIPA buffer+2× Protease Inhibitors (Roche, #4693159001). The protein concentration was determined with the Pierce™ BCA Protein Assay Kit (Thermo Fisher Scientific, #23225) according to the manufacturer's protocol. Equal amounts of lysates were loaded on a 14% or 18% SDS-Page for size fractionation. After electrophoresis, proteins were immunoblotted on a PVDF-membrane (Sigma-Aldrich, #3010040001) for 1 h at 100 V and blocked with 5% milk in Tris-buffered saline and 1% Tween20. Proteins were detected with rabbit anti-ARPC5 (Novus, #NBP2-67350, lot #HL0702, 1:1000), rabbit anti-ARPC5L (Abcam, #ab169763, lot #GR121833-4, 1:2000) or mouse anti-ARPC3 (anti-Arp2/3 Complex Antibody Clone 13c9, Millipore, #MILL-MABT95, lot3283230, 1:5000). HRP-conjugated goat anti-rabbit IgG (Cell Signaling Technology, #7074S, 1:3000) and HRP mouse IgG kappa binding protein (Santa Cruz, sc-516102, 1:3000) were used as secondary antibodies before detection with ECL chemiluminescent substrate (Cell Signaling Technology, #12630S, 1:1). Anti-Tubulin (Proteintech, #HRP-66031, 1:5000) was used as a loading control and detected as a 55 kDa band.

### Immunofluorescence analysis

Cells were seeded on coverslips in a 12-well plate and incubated overnight in RPMI 1640 supplemented with 10% FBS and 1% P/S. Cells plated on coverslips were incubated with 4% paraformaldehyde for 10 mins at room temperature and washed three times with 1× PBS. Cells were then incubated with 0.5% Triton X-100 for 2 mins at room temperature and washed three times with 1× PBS. Coverslips were incubated with blocking buffer for 30 mins at room temperature. Coverslips were then incubated with mouse anti-vinculin antibody (Sigma-Aldrich, V4505, batch #0000216740, 1:500) overnight at 4°C. Coverslips were then washed three times with 1× PBS and incubated with Alexa Fluor 488-conjugated anti-mouse antibody (Thermo Fisher Scientific, A21202, 1:500) and Alexa Fluor 647-conjugated Phalloidin (Thermo Fisher Scientific, A22287, 1:500) for 30 mins at room temperature. After three washes in 1× PBS, cells were incubated with DAPI (Cell Signaling Technology, 4083S, 300 nM) for 5 mins. Coverslips were then washed twice with 1× PBS and once with distilled water before mounting the coverslips onto microscopy slides using 4 µl Mowiol. Coverslips were imaged on a Zeiss Axioplan2 microscope with a 63×/1.4 NA Plan-Achromat objective and a Photometrics Cool Snap HQ cooled charge-coupled device camera. Data for 20-30 cells per sample were analyzed using FIJI.

### Random migration assay

Hela WT and ARPC5 KO cells were seeded in 24-well glass bottomed plates (Cellvis) at 1×10^4^ cells/well in 500 µl RPMI 1640 supplemented with 10% FBS and 1% P/S. Cells were imaged and analyzed using the Livecyte system (Phasefocus) every 10 mins for 24 h at 10× with two fields of view per well. The imaging chamber was maintained at 37°C with 5% CO_2_.

### Wound healing assay

HeLa and MDA-MB-231 WT and ARPC5 KO cells were seeded in a 96-well ImageLock plate (Sartorius, #4806) and incubated for 24 h. The following day, cells were scratched using the Incucyte^®^ WoundMaker 96 (Sartorius). Cells were washed twice with PBS and cell migration towards the scratch was monitored with Sartorius Incucyte S3 System for 72 h, with one image obtained per h under brightfield 10× magnification. Images were analyzed using IncuCyte 2019B Rec2 software. The relative scratch intensity was calculated as %RWD(t)=[(wt-w0)/(ct-c0)]×100.

### Under agarose migration assay

Glass-bottomed 8-well µ-Slides (ibidi) were coated with human ICAM-1 (3 µg/ml; R&D Systems) overnight at 4°C. The wells were then washed and blocked with 2% fatty acid-free bovine serum albumin (BSA; Sigma-Aldrich) for 20 min at room temperature. Agarose gel mixture was prepared by mixing 2× phenol red-free HBSS, RPMI supplemented with 20% FBS+2% P/S+4 mM L-glutamine+2× NEAA+2× sodium pyruvate+50 mM HEPES+50 µM β-mercaptoethanol and 2% liquid UltraPure Agarose (Thermo Fisher Scientific) in 1:2:1 ratio. The mixture was shaken at 300 rpm at 56°C for 10 mins. Before adding the mixture to the wells, human CXCL12 (250 ng/ml; Peprotech) or vehicle control was added to the gel mixture. Gel mixtures were then added to the wells accordingly and allowed to solidify and cool for at least 1 h. Jurkat WT and ARPC5 KO cells were labeled with CTV or CFSE (Thermo Fisher Scientific) according to the manufacturer's protocol before imaging. Propidium iodide was added at 3 µM to identify dead cells. Approximately 6×10^4^ cells in 10 µl were injected into an agarose gel matrix. Cells were then imaged using an inverted widefield Nikon Ti2 Eclipse long-term time lapse system with an LED illumination system. The imaging chamber was maintained at 37°C with 5% CO_2_. A Nikon 20× Ph2 planar (plan) apochromatic (apo) (0.75 N.A.) air objective was used to acquire images at a single *z*-slice every 15 s for 10 min with three fields of view per well. Cell shape and migration parameters were analyzed using FIJI plug-in TrackMate.

### CXCL12-induced F-actin polymerization assay

Jurkat and THP1 WT and ARPC5 KO cells were seeded in 96-well plate and stimulated with RPMI 1640+100 ng/ml CXCL12 (Peprotech, 300-28A) or RPMI 1640 for the indicated time points. Cells were fixed with ice-cold 4% paraformaldehyde (PFA) for 20 min and permeabilized with 0.1% Triton X-100 for 10 min. To detect F-actin, cells were incubated with Alexa Fluor 647-Phalloidin (Cell Signaling Technology, #8940S, 1:20) for 20 min. Mean fluorescence intensity (MFI) was measured using BD LSRFortessa™ and data were analyzed using FlowJo (Version 10.8.1). Increase in MFI was calculated as a percentage using the following formula, with MFI of the unstimulated samples defined as 100% (Narumi, 2022): 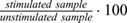.

### Generation of conditional mouse models

Mice were bred and maintained in a specific pathogen-free room with a 12 h light/12 h dark cycle, and access to *ad libitum* food and water. The conditional *Arpc5* KO strain was generated by the Crick Genetic Modification Service using a CRISPR-Cas9 approach in embryonic stem cells (ESCs) with gRNA-1, 5′-TAGTTTCAGTATAAAGTCTA-3′ and gRNA-2, 5′-CCCTTTAAAGCTGGACGTGG-3′, which introduce the two 5′ and 3′ loxP sites within intron 1-2 and intron 2-3, respectively. Removal of exon 2 induces a frameshift and an exogenous stop codon within exon 3, resulting in only the first 55 residues of ARPC5 being expressed. The two Cas9-gRNA-Puro plasmids were generated by inserting the CRISPR-Cas9 target sequences into PX459 plasmid (a gift from Feng Zhang; Ran et al., 2013; Addgene plasmid #48139). The pMA donor plasmid contained a synthesized 734 bp fragment corresponding to exon 2 floxed by two loxP sites and 1 kb flanking homology arms (GeneArt, Thermo Fisher Scientific). Sequence-verified Cas9-gRNA-Puro plasmids and pMA donor plasmid were co-transfected into in-house C57BL6/N 6.0 ESC using Lipofectamine 2000 (Thermo Fisher Scientific) before selection with 2 µg/ml puromycin for 48 h. Putative targeted clones were initially identified by reverse transcription quantitative polymerase chain reaction (RT-PCR) assays on genomic DNA using gene-specific probes designed by Transnetyx to detect the integration of 5′ and 3′ loxP sites. Integrity of the loxP integrations was confirmed by Sanger sequencing of PCR amplicons. Correctly targeted ESC clones were microinjected into blastocysts derived from albino C57BL/6J mice [B6(Cg)-Tyrc-2J/J; strain #000058, The Jackson Laboratory) and then transplanted into the uteri of pseudo-pregnant CD1 females. The resulting chimeras were crossed to albino C57BL/6J, and their offspring were initially screened for the integration of the loxP sites by amplicon sequencing. Heterozygous floxed offspring were validated by Sanger sequencing across a 3.2 kb amplicon encompassing the targeted allele. Two founder strains were maintained on a C57BL/6J background by backcrossing more than three times. Constitutive *Arpc5* KO mice were obtained by mating Arpc5 floxed mice with *Pgk1-cre* [B6.C-Tg(Pgk1-cre)1Lni/CrsJ; Lallemand et al., 1998]. All mice were genotyped at weaning age at Transnetyx as described above.

### Study approval

All animal work was authorized by UK Home Office project license P7E080263 and personal licenses following the approval by the Animal Welfare and Ethical Review Body of The Francis Crick Institute. Patients were registered in the Iranian Primary Immunodeficiency Registry (IPIDR) at the Immunology, Asthma & Allergy Research Institute (IAARI). The Ethical Committee of IAARI approved this study (#IR.TUMS.IAARI.REC.1397.002), and written informed consent for participation in this study was obtained from their parents.

### Whole-mount embryo immunofluorescence and volume imaging

Timed-pregnant females were used to harvest staged embryos based on the date of vaginal plug as 0.5 dpf. Embryos up to 13.5 dpf were dissected to examine the morphology under a Leica MZ16 stereomicroscope equipped with Micropublisher 6 Colour Camera (Teledyne Photometrics). The yolk sacs were harvested for genotyping by PCR with the following primer pairs: Arpc5-1 For, CCAGAATAATCAGCCAGCATTTCAG, and Arpc5-1 Rev, CTTGTCACAAGCTCCCTTTAAAGC, which generates an 819 bp unexcised product and 119 bp excised product for the Arpc5 allele; Cre For, TCATCTCCGGGCCTTTCG, and Cre Rev, GACAGAAGCATTTTCCAGGTATGC, which yields a 200 bp product for Cre transgene. Dissected embryos were fixed with cold 4% PFA in PBS overnight at 4°C. The embryos were washed with PBS containing 0.1% Triton X-100 (PBST) three times for 1 h each, and then incubated with PBST containing 2% BSA (blocking buffer) for 4 h at room temperature. The embryos were subsequently incubated overnight at 4°C in solution containing Cy3-conjugated α-smooth muscle actin monoclonal antibody (Merck, C6198, batch #0000122897, 1:100) and DAPI. Embryos were washed three times with PBST for 1 h and mounted in 1% agarose solution before imaging. *Z*-stack tiled images were obtained using an inverted Zeiss 880 confocal microscope and Plan-Apochromat 10×/0.45 M27 air objective at 1024×1024 pixels with a step of 5 µm. The images were stitched and adjusted for intensity using Zeiss Zen Blue 2.6 software.

### Statistical analysis

Statistical significance was calculated using a paired ratio *t*-test, an unpaired *t*-test, two-way ANOVA or one-way ANOVA with Tukey's correction using GraphPad Prism 6.0 software. A *P*-value of <0.05 was considered statistically significant (*P<0.05; **P<0.01; ***P<0.001; ****P<0.0001).

### RT-PCR

Total RNA from cells was extracted using the RNeasy Mini Kit (Qiagen) according to the manufacturer's instructions. cDNA was generated using 1 μg RNA using M-MLV Reverse Transcriptase (Promega) according to the manufacturer's protocol. RT-PCR was performed using SYBR Green Dye (Applied Biosystems) on the StepOne Plus system (Applied Biosystems). Results were normalized to the tissue housekeeping gene *SDHA*. Relative expression levels were calculated as 2^[Ct(gene)−Ct(SDHA)]^.

## Supplementary Material

10.1242/dmm.050145_sup1 Supplementary information
