# Supplementary material for: ARPC5 deficiency leads to severe early-onset systemic inflammation and mortality
Source: Dis Model Mech. 2023 Jul 21;16(7):dmm050145. doi: 10.1242/dmm.050145 (PMC10387347; doi:10.1242/dmm.050145)
Supplement: Supplementary information [file dmm-16-050145-s1.pdf]

A

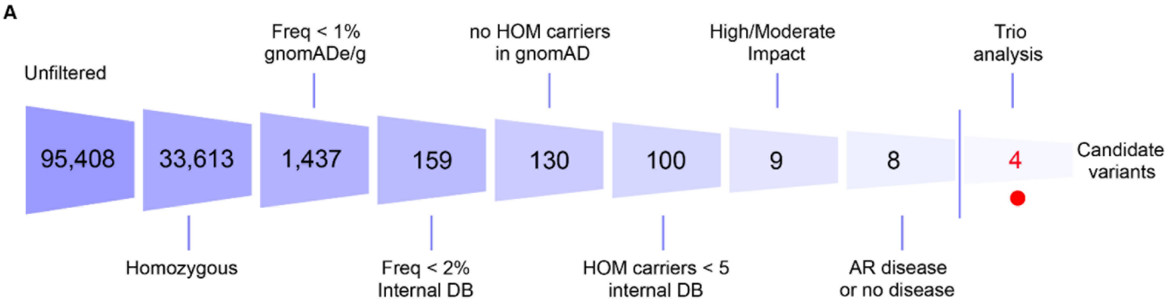

B

	Postion	Gene	Var. type DNA	Var. type Prot	Impact	Transcript
●	1-183633108	ARPC5	deletion	frameshift	HIGH	ENST00000294742.6
	6-41687981	TFEB	point mutation	missense	MODERATE	ENST00000358871.6
●	8-6691914	MCMDC2	point mutation	missense	MODERATE	ENST00000422365.7
●	8-74010085	LY96	point mutation	missense	MODERATE	ENST00000284818.6
	15-89621528	TICRR	point mutation	missense	MODERATE	ENST00000268138.11
●	18-70024725	RTTN	point mutation	missense	MODERATE	ENST00000640769.1
	6-4116028	ECI2	delins	splice_region_variant	MODERATE	ENST00000380118.7
	17-41149517	KRTAP4-5	insertion	protein_altering_variant	MODERATE	ENST00000343246.6

CDS change	Protein change	Count gnomad exom Het	Count gnomad exom HOM
c.198del	p.Ile67SerfsTer8	NA	NA
c.639G>T	p.Gln213His	NA	0
c.2018G>A	p.Gly673Glu	2	0
c.287G>A	p.Arg96Gln	25	0
c.3290C>T	p.Ser1097Leu	56	0
c.5947A>G	p.Asn1983Asp	NA	NA
c.1030-3_1031delinsTAGGT	p.Thr84delinsMetProProGlnLeuPro	NA	NA
c.250_251ins15	NA	NA	NA

Fig. S1. WES variant filtering strategy for index patient V1.5 (**A**) Diagram of the filtering strategy employed for the WES analysis of the index (VI.5) patient (AF = Allele Frequency; 1KG = Thousand Genome Project, https://www.internationalgenome.org/; GnomAD = Genome Aggregation Database, https://gnomad.broadinstitute.org/; (**B**) List of homozygous rare variants present in VI.4 and heterozygous in both parents. This list was filtered as detailed in the Material and Methods.

Disease Models & Mechanisms • Supplementary information

A

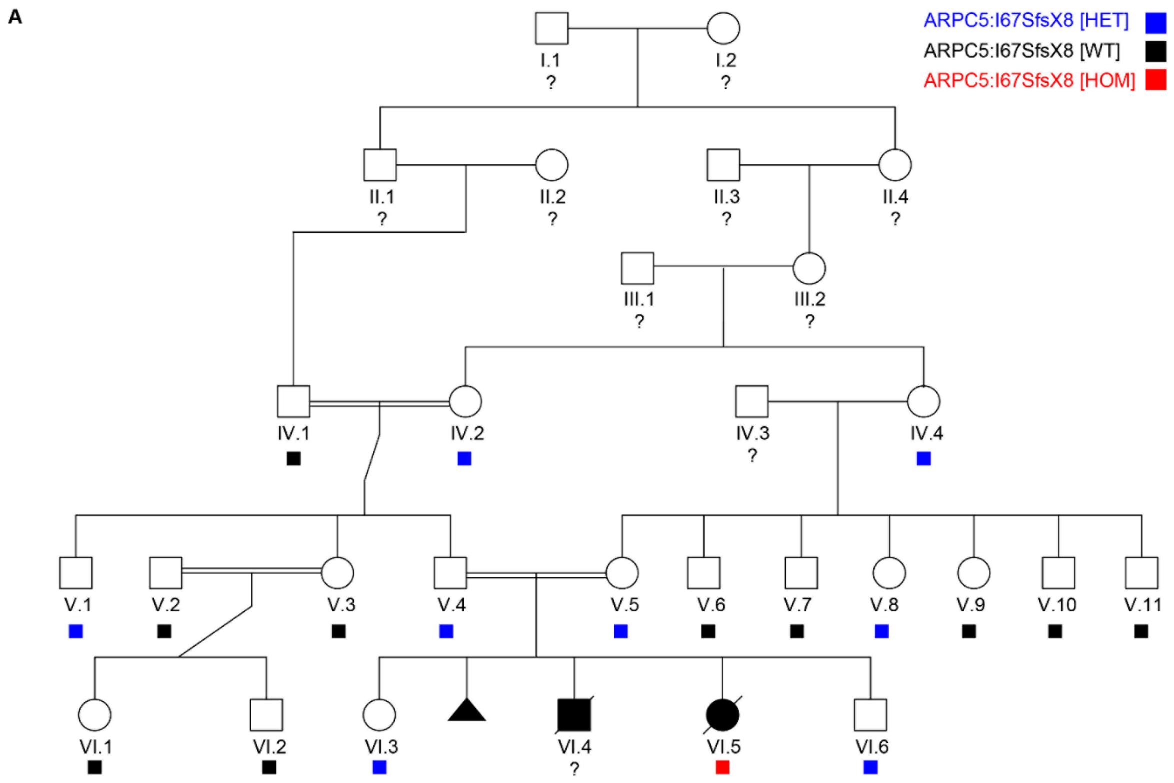

Fig. S2. Extended index family pedigree showing *ARPC5* variant segregation Pedigree of the extended family showing the segregation of the c.189delT variant in *ARPC5* identified in VI.5.

Disease Models & Mechanisms • Supplementary information

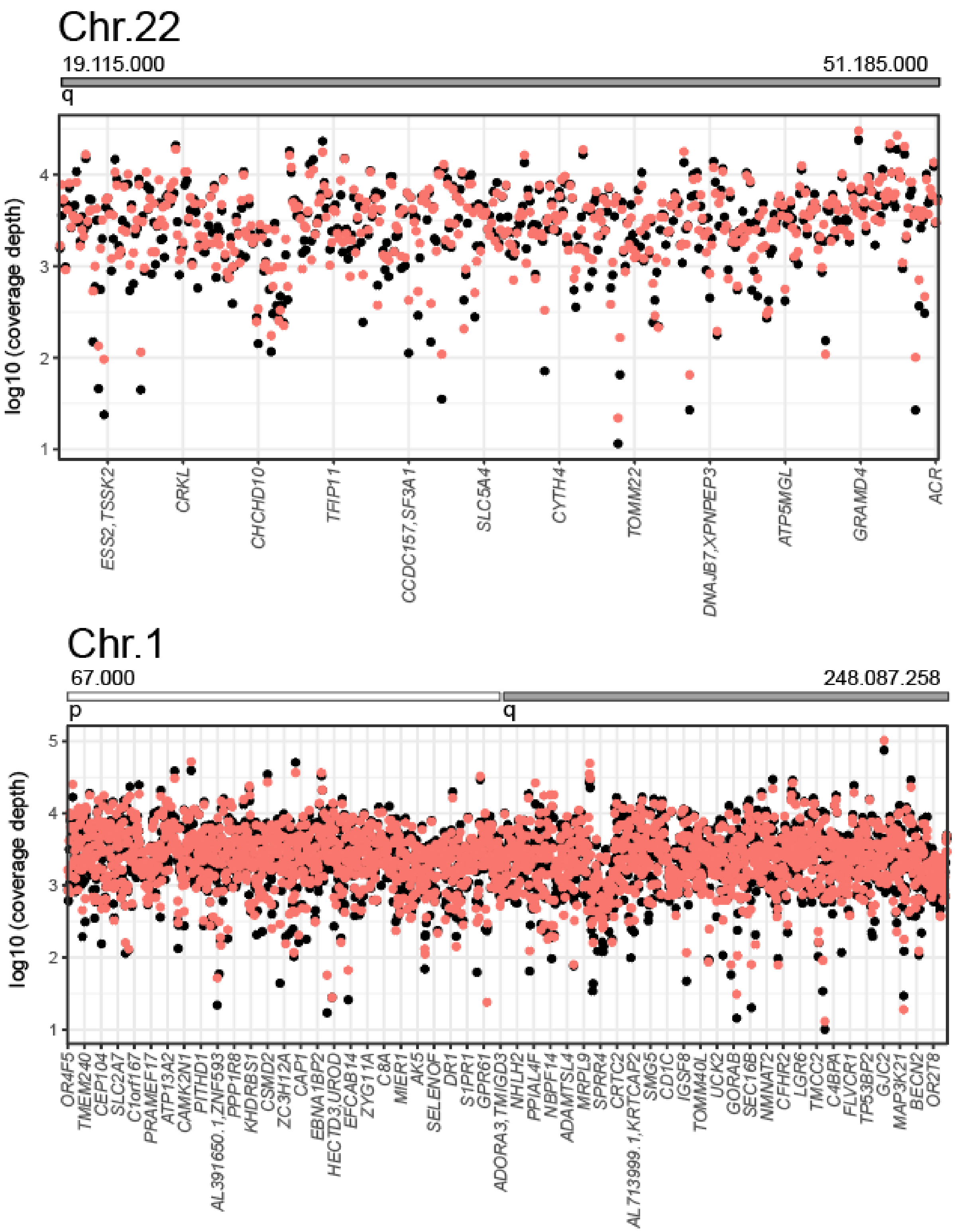

Fig. S3. Coverage analysis of Chr.22 showing log10 of normalized coverage (y Axis) of V1.5 (red) compared to the mean of other 18 family members (black). Chr.1 is also shown as additional control. Each dot represents one gene; in the x axis the name of only a subset of genes is displayed.

Disease Models & Mechanisms • Supplementary information

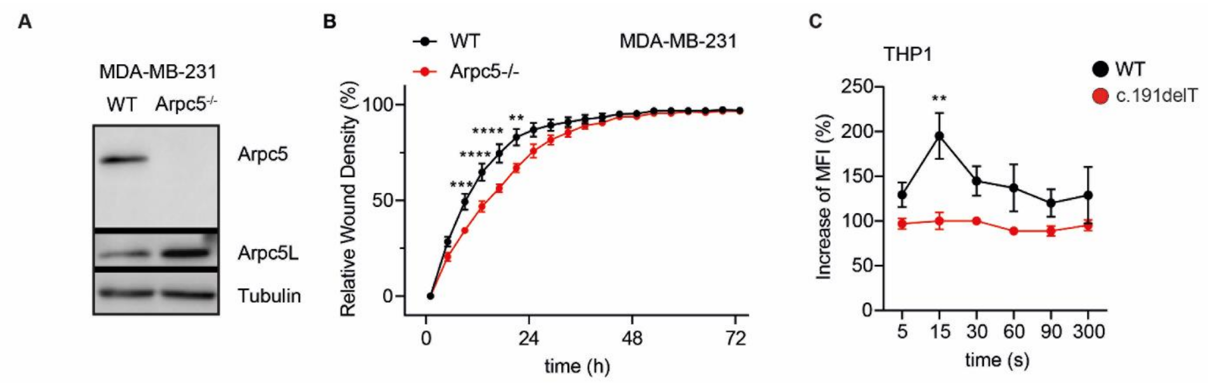

Fig. S4. ARPC5 deficiency affects actin cytoskeleton organization and function. (A) Confirmation of ARPC5 deficiency in MDA-MB-231 cells via Western Blot. **(B)** Relative wound density of MDA-MB-231 WT and *ARPC5*$^{-/-}$-cells. (**C**) F-actin polymerization in c.191delT-THP1cells with or without ARPC5 at the indicated time point after stimulation with CXCL12. Three independent experiments were analyzed. Statistical analysis was performed using a Two-way ANOVA. **, ***, **** indicates respectively $p < 0.01$, $p < 0.001$ and $p < 0.0001$; error bars indicate standard deviations; MFI = Mean Fluorescence Intensity.

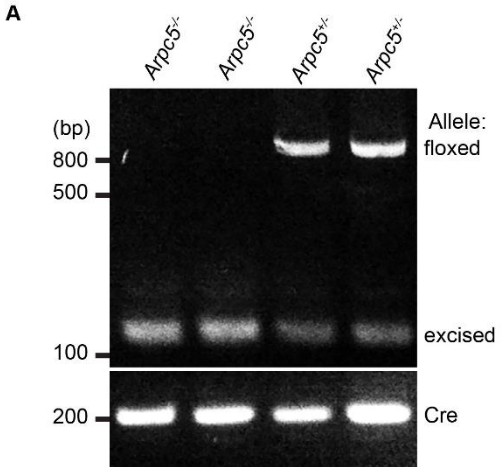

Fig. S5. Arpc5 deficiency in mice results in defective organogenesis and embryonic lethality Genotyping PCR showing the floxed and excised allele of *Arpc5*, as well as *Cre* transgene.

Disease Models & Mechanisms • Supplementary information
